# Commentary: Voltage Gating of Mechanosensitive PIEZO Channels

**DOI:** 10.3389/fphys.2018.01565

**Published:** 2018-11-20

**Authors:** Lars Kaestner, Stephane Egee

**Affiliations:** ^1^Theoretical Medicine and Biosciences, Saarland University, Homburg, Germany; ^2^Experimental Physics, Saarland University, Saarbrücken, Germany; ^3^Sorbonne Université, CNRS, UMR 8227, Integrative Biology of Marine Models, Station Biologique de Roscoff, Roscoff, France; ^4^Laboratoire d'Excellence GR-Ex, Paris, France

**Keywords:** erythrocyte, non-selective voltage-dependent cation channel, Piezo1, Hereditary Xerocytosis, patch-clamp

This is a commentary about a recently published paper, elucidating the voltage dependent properties of a channel that was primarily regarded as a mechanosensitive channel: Piezo1 (Moroni et al., 2018). Here we consider the importance of this report in the red blood cell field and provide a link to previously reported data on functional channel activity in red blood cells.

Piezo1 is an ion channel that is believed to be present in red blood cells. Although data from molecular biology are limited (Kaestner, 2015) mutations in Piezo1 cause the red blood cell-related disease Hereditary Xerocytosis (Zarychanski et al., 2012; Bae et al., 2013), which provides convincing evidence. Beside this pathophysiological scenario, the interplay of Piezo1 and the Gardos channel appear to have a vital physiological function in volume adaptation when red blood cells pass constrictions in the narrowest of the capillaries or the spleen slits (Faucherre et al., 2014; Cahalan et al., 2015; Danielczok et al., 2017).

Among the ion channels in red blood cells there are reports about a non-selective voltage-dependent cation channel (Christophersen and Bennekou, 1991; Kaestner et al., 1999; Rodighiero et al., 2004). This channel is also permeable to Ca^2+^ and comprises a rather unique hysteresis like open probability (Kaestner et al., 2000). Although the physiological function of voltage activated ion channels in non-excitable cells, such as red blood cells is everything else but obvious, a proposal of their regulation was recently published (Kaestner et al., 2018). However, so far the molecular identity of this channel remained obscure (Kaestner, 2011; Bouyer et al., 2012).

The very recent report about voltage-gating of Piezo channels (Moroni et al., 2018) provides evidence that the molecular identity of the non-selective voltage-dependent cation channel in red blood cells might be Piezo1. Figure 1 shows single channel currents of the non-selective voltage-dependent cation channel recorded in red blood cells (Figure 1A) and of Piezo1 overexpressed in Neuro2A cells (Figure 1B). There is quite some similarity in the channel properties: The single channel conductance in Figures 1A,B is 21 ± 5 and 27.1 ± 1.2 pS, respectively. The general dynamic behavior is similar and in both recordings, substates of the channel activity can be seen. The recordings show a slightly different gating, which very well could be caused by differences in the bilayer composition of the plasma membrane between red blood cells and Neuro2A cells. Beside the different cell types one should consider that both recordings (Figure 1A vs. Figure 1B) originate from different laboratories (although performed in the same city) with different equipment and vastly different experimental protocols (including recording solution, patch-clamp configuration and voltage protocols). Furthermore, we like to mention that the different appearance of the traces is more due to different sampling frequencies and different filtering in both recordings than due to channel properties. More importantly, the hysteresis like behavior of the open probability of the non-selective voltage-dependent cation channel recorded in red blood cells is depicted in Figure 1C. A similar pattern could be achieved when Piezo1 was measured with or without a previous conditioning step (Figure 1D). Although the hysteresis like gating is a unusual property, mathematical simulation can well explain the phenomenon (Andersson, 2010).

**Figure 1:**
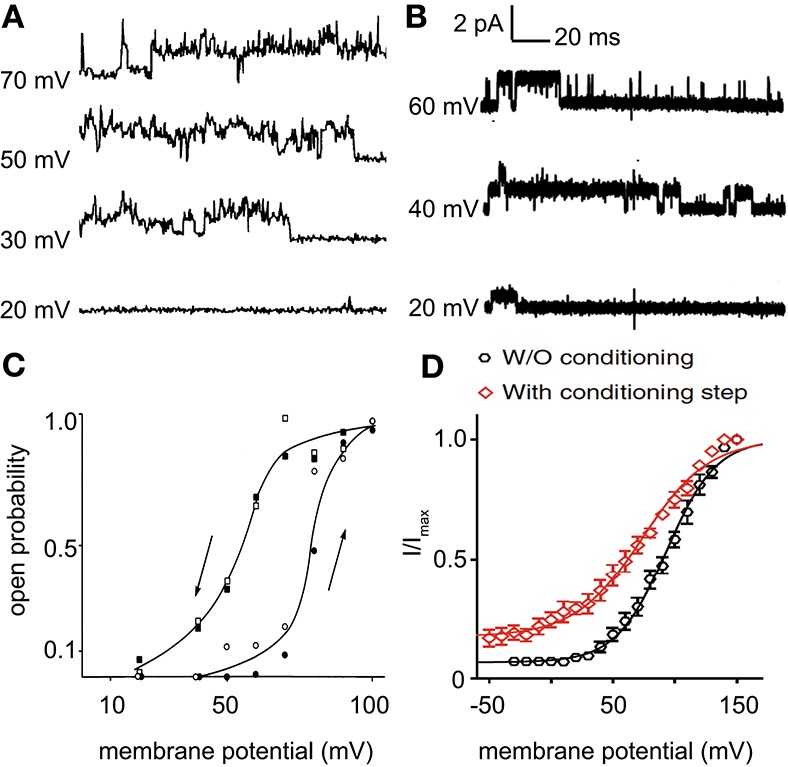
Comparison of the non-selective voltage activated cation channel recorded in red blood cells **(A,C)** and Piezo1 recorded in overexpressing Neuro2A cells **(B,D)**. **(A)** Current traces of the non-selective voltage-dependent cation channel in inside-out patches of red blood cells in Na-tartrate-solution in mM (bath solution: 70 Na-tartrate, 2.5 BaCl_2_, 10 MOPS, 10 glucose, 75 saccharose, pH = 7.4; pipette solution: 20 Na-tartrate, 2.5 BaCl_2_, 10 MOPS, 10 glucose, 225 saccharose, pH = 7.4). **(B)** Current traces of Piezo1 in outside-out patches of overexpressing Neuro2A cells in symmetrical NaCl-solution in mM (140 NaCl, 10 HEPES, 5 EGTA, pH = 7.4). The conductance of the channels presented in **(A,B)** is 21 ± 5 and 27.1 ± 1.2 pS, respectively. **(C)** The open state probability as function of the membrane potential. In both series (open symbols and filled symbols), the open probability was calculated from 3 min of continuous recording at each potential. The curves were drawn by eye. **(D)** Tail currents from individual cells were normalized to their maximum and fitted to a Boltzmann relationship. Pooled data are shown as mean ± SEM. **(A,C)** are reproduced from Kaestner et al. (1999, 2000), respectively and **(B,D)** from Moroni et al. (2018).

Although current traces have a certain fingerprint, comparison of Figure 1A with Figure 1B does not provide evidence that the non-selective voltage-dependent cation channel in red blood cells is Piezo1 but it is compatible with this hypothesis. More interesting is the unusual hysteresis like behavior plotted in Figures 1C,D. In this respect Moroni et al. provide the explanation that positive voltage together with outward permeation may hold the channel in a fully active conformation preventing the desensitized state (Moroni et al., 2018). This statement is 100% compatible with the recordings performed on red blood cells, where the direction of the membrane potential change (indicated by the arrows in Figure 1C) provides rational explanation to the difference in the open probability. This direction of the membrane potential change goes hand in hand with the amount of outward permeating ions. Under physiological conditions in red blood cells, the non-selective voltage-dependent cation channel is thought to be closed at membrane potentials below +40 to +30 mV. This may imply that regarding very fast closing of the channel in patch-clamp may be accounted for by the mechanosensitivity and voltage-dependence since in red blood cells deactivation has a time constant of below 15 ms (Bennekou et al., 2003).

With the recent paper reporting voltage gating of mechanosensitive Piezo channels, we got a new and very plausible indication for the molecular identity of the non-selective voltage-dependent cation channel in red blood cells. This may explain why sudden calcium entry through Ca^2+^ permeating mechanosensitive channel may induce longer permeation and thus enhanced subsequent Gárdos channel activity that eventually lead to phenotypic xerocytosis (Dyrda et al., 2010) since Ca^2+^ entry will lead to transient depolarisation.

A further detailed study on red blood cells is required to confirm the observations performed in overexpressing cells (Moroni et al., 2018) also in the primary red blood cells. The application of planar patch-clamp chips may technically favor the investigation of the rather difficult to patch red blood cells (Minetti et al., 2013; Rotordam et al., 2018).

To know about the voltage sensitivity of Piezo1, will allow (independent whether identical with the non-selective voltage-dependent cation channel in red blood cells or not) a differential view of the role of Piezo1 and its interaction with other ion channels especially in red blood cells in health and disease (Thomas et al., 2011; Kaestner et al., 2018). This is of particular importance since an increasing number of Piezo1 mutations have been reported (Zarychanski et al., 2012; Albuisson et al., 2013; Andolfo et al., 2013; Bae et al., 2013) and also other channelopathies, e.g., involving the Gardos channel may interact with the non-selective voltage-dependent cation channel/Piezo1 (Glogowska et al., 2015; Rapetti-Mauss et al., 2015, 2016; Fermo et al., 2017; Kaestner et al., 2018).

## Author contributions

Both authors have made a substantial, direct and intellectual contribution to the work, and approved it for publication.

### Conflict of interest statement

The authors declare that the research was conducted in the absence of any commercial or financial relationships that could be construed as a potential conflict of interest.
